# Letter to the editor: risk comorbidities of COVID-19 in Parkinson’s disease patients in Germany

**DOI:** 10.1186/s42466-020-00069-x

**Published:** 2020-08-11

**Authors:** Daniel Richter, Dirk Bartig, Christos Krogias, Lars Tönges

**Affiliations:** 1 Department of Neurology, St. Josef-Hospital, Ruhr University Bochum, Gudrunstr. 56, 44791 Bochum, Germany; 2 DRG Market, Osnabrück, Germany; 3 Neurodegeneration Research, Protein Research Unit Ruhr (PURE), Ruhr University Bochum, Bochum, Germany

**Keywords:** COVID-19, Parkinson’s disease, Infection risk

## Main text

The current coronavirus disease 2019 (COVID-19) pandemic has global significance. From a neurological point of view, COVID-19 patients frequently present with neurological symptoms [1]. Moreover, patients with chronic neurological diseases such as Parkinson’s disease (PD) are particularly challenged as this pandemic affects PD patient care, routine visits, medical supply issues and causes disruption to PD research [2]. Most importantly, PD patients often fall into the core population which is at risk for COVID-19. The risk profile for COVID-19 is confirmed very specifically in recent data from an own analysis of PD patients in Germany [3].

We here analyzed current data of all inpatient PD patients in Germany as coded in the German Diagnosis-Related Groups (G-DRG) system which is relevant for reimbursement of inpatient treatment cost. For the year 2018, we extracted the data of PD patients to determine the presence of comorbidities with known high risk for a severe course of COVID-19 [4] such as hypertension, cardiovascular disease, cerebrovascular disease, diabetes, hepatitis B infection, chronic obstructive pulmonary disease, and chronic kidney disease. We also analyzed the proportion of PD patients who are in need of care measures. Overall, this analysis provides data of 45.345 inpatient cases with primary diagnosis PD in the year 2018.

We found a strikingly high prevalence of these risk comorbidities in the PD cohort, especially in the patient subgroup without presence of motor fluctuations. This specifically supports the hypothesis that PD patients in general and some subgroups could be at a higher risk for a severe course of COVID-19. Moreover, we found a high level of care dependency of PD patients which makes it impossible to completely limit contacts to other people or self-isolate because of their need for care staff (Table 1).

**Table 1:** Percentage of comorbidities with assumed high risk for a severe course of COVID-19^5^ and care dependency given for all inpatient cases of PD (*n* = 46,345) in Germany from the year 2018

ICD-10-GM	G20.00	G20.01	G20.10	G20.11	G20.20	G20.21	G20.90	G20.91
**Absolute number of PD cases**	3164	2387	10,499	20,266	1541	4303	3046	1139
**Percentage with comorbidity (ICD-Code)**								
Hypertension (I10)	53.60%	49.27%	53.07%	47.42%	51.01%	44.97%	52.53%	51.54%
Cardiovascular disease (I25)	11.71%	9.64%	13.21%	11.37%	15.64%	11.71%	14.71%	13.96%
Cerebrovascular disease (I69)	2.47%	1.59%	3.57%	2.68%	5.58%	4.04%	4.53%	3.42%
Diabetes type 2 (E11)	16.59%	14.03%	18.16%	14.28%	18.36%	14.66%	20.88%	20.63%
Chronic viral hepatitis B or C (B18)	0.09%	0.0%	0.09%	0.08%	0.0%	0.0%	0.0%	0.0%
COPD (J44)	2.75%	1.93%	3.02%	2.62%	3.50%	2.46%	3.32%	2.63%
Chronic kidney disease (N18)	7.84%	6.07%	11.55%	9.42%	14.60%	12.36%	14.05%	14.22%
**Percentage with care dependency (ICD-Code)**								
Care dependency^a^ (Z74)	14.60%	18.64%	32.57%	29.03%	62.95%	49.06%	42.35%	40.65%

Parkinson’s disease diagnoses of the ICD 10th revision, German modification (ICD-10-GM): **G20.00 =** Primary Parkinson’s syndrome without or with less impairment and no fluctuation; **G20.01 =** Primary Parkinson’s syndrome without or with less impairment and fluctuation; **G20.10 =** Primary Parkinson’s syndrome moderate to severe impairment and no fluctuation; **G20.11 =** Primary Parkinson’s syndrome moderate to severe impairment and fluctuation; **G20.20 =** Primary Parkinson’s syndrome with the most serious impairment and no fluctuation; **G20.21 =** Primary Parkinson’s syndrome with the most serious impairment and fluctuation; **G20.90 =** Primary Parkinson’s syndrome not further defined and no fluctuation; **G20.91 =** Primary Parkinson’s syndrome not further defined and fluctuation

*COPD* chronic obstructive pulmonary disease, *ICD* International Statistical Classification of Diseases and Related Health Conditions

^a^Care dependency including dependency due to impaired mobility, dependency for personal hygiene and need for constant supervision

We additionally analyzed the inpatient prevalence of non-aspiration and aspiration pneumonia in which PD was coded only as secondary diagnosis to differentiate pneumonia subtypes [3]. Interestingly, non-aspiration pneumonia was the most common admission primary diagnosis (8256 cases) while aspiration pneumonia was coded to a less numerous extent (3777 cases; data not shown). This indicates that aspiration is not the most common cause for pneumonia in PD. In clinical practice, the differentiation between aspiration and non-aspiration pneumonia may be difficult because silent aspiration can also occur in patients with swallowing disorders such as PD and there is no precise diagnostic to differentiate the two entities. Importantly, the more frequently coded non-aspiration pneumonia in PD represents a spectrum of diseases including bacterial and fungal but also viral pneumonias. Thus, we consider PD patients to have an increased risk also for viral pneumonias including Severe Acute Respiratory Syndrome Coronavirus 2 (SARS-CoV-2).

In summary, this nationalwide data from Germany points out several clinical characteristics of inpatient PD patients that are risk factors for a more severe course of COVID-19. Apart from the increased occurrence of pulmonary infections, strict social distancing is often difficult due to care dependency. We do not consider PD itself to represent a risk factor for COVID-19 and a diagnosis of PD should not lead to any difference in decisions making to treat COVID-19 in these patients. The frequent comorbidities with PD rather highlight the importance of preventive strategies such as telemedicine consultations and preventive testing of care staff. Neurologists and movement disorders specialists should consider a very careful attitude in the treatment of Parkinson’s patients with prudent prevention of infections in these challenging times.

## Data Availability

The G-DRG datasets analysed during the current study are available online from the Federal Statistical Office (DRG-statistic 2018, Federal Statistical Office, www.destatis.de).

## Abbreviations

COVID-19: Coronavirus disease 2019
G-DRG: German Diagnosis- Related Groups
PD: Parkinson’s disease
SARS-CoV-2: Severe Acute Respiratory Syndrome Coronavirus 2
